# Spin-preserving chiral photonic crystal mirror

**DOI:** 10.1038/s41377-020-0256-5

**Published:** 2020-02-20

**Authors:** Behrooz Semnani, Jeremy Flannery, Rubayet Al Maruf, Michal Bajcsy

**Affiliations:** 1 Institute for Quantum Computing (IQC), University of Waterloo, Waterloo, N2L3G1 ON Canada; 2 Department of Electrical & Computer Engineering, University of Waterloo, Waterloo, N2L3G1 ON Canada; 3 Department of Physics and Astronomy, University of Waterloo, Waterloo, N2L3G1 ON Canada

**Keywords:** Nanophotonics and plasmonics, Photonic crystals

## Abstract

Chirality refers to a geometric phenomenon in which objects are not superimposable on their mirror image. Structures made of nanoscale chiral elements can exhibit chiroptical effects, such as dichroism for left- and right-handed circularly polarized light, which makes these structures highly suitable for applications ranging from quantum information processing and quantum optics to circular dichroism spectroscopy and molecular recognition. At the same time, strong chiroptical effects have been challenging to achieve even in synthetic optical media, and chiroptical effects for light with normal incidence have been speculated to be prohibited in thin, lossless quasi-two-dimensional structures. Here, we report an experimental realization of a giant chiroptical effect in a thin monolithic photonic crystal mirror. Unlike conventional mirrors, our mirror selectively reflects only one spin state of light while preserving its handedness, with a near-unity level of circular dichroism. The operational principle of the photonic crystal mirror relies on guided-mode resonance (GMR) with a simultaneous excitation of leaky transverse electric (TE-like) and transverse magnetic (TM-like) Bloch modes in the photonic crystal slab. Such modes are not reliant on the suppression of radiative losses through long-range destructive interference, and even small areas of the photonic crystal exhibit robust circular dichroism. Despite its simplicity, the mirror strongly outperforms earlier reported structures and, contrary to a prevailing notion, demonstrates that near-unity reflectivity contrast for opposite helicities is achievable in a quasi-two-dimensional structure.

## Photonic crystals: chiral mirrors

Chiral mirrors that selectively reflect either left- or right-handed circularly polarized light are potentially useful for applications in quantum optics where the spin state (helicity) of light needs to be controlled. Now, Behrooz Semnani and coworkers from the University of Waterloo in Canada have built such mirrors from a thin (thickness of 309nm) silicon nitride photonic crystal membrane featuring an array of circular and ellipitical holes arranged in a carefully-designed pattern. Operating at 870nm in the infrared, the mirrors make use of a guided-mode resonance of transverse electric and transverse magnetic modes. Upon illumination, light with the chosen spin is almost perfectly reflected from the mirror with its state of polarization preserved while light with the opposite spin is completely transmitted. Polarization-resolved imaging experiments demonstrate the behaviour of the chiral mirrors.

## Introduction

An ultrathin spin-preserving chiral mirror that completely reflects only one spin state of light upon normal illumination without reversing the light’s handedness is a chiroptical structure of particular interest, as metallic, dielectric-stack, and even Faraday mirrors flip the helicity (i.e., the spin) of light upon reflection. In addition, Fabry–Pérot cavities made of spin-preserving mirrors would exhibit a variety of unique properties^1^, such as formation of null-free resonant modes. Such self-polarizing “chiral cavities” formed by these thin mirrors will open up tantalizing possibilities in quantum optics^2–5^ and optomechanics, with opportunities ranging from the realization of novel types of gas lasers based on mesoscale fiber-integrated cavities^6^ to fundamental studies of light–matter interactions in systems, such as membrane-in-the-middle coupled cavities^7^. A particular advantage of self-polarizing chiral cavities is the relatively high strength of optical transitions between atomic levels coupled by circularly polarized light compared with levels coupled by linearly polarized light. In addition, such optical transitions with circularly polarized light are often effectively closed and allow the isolation of a two-level system from the generally complicated level structure of commonly used atoms.

However, the realization of an ultrathin chiral mirror that completely reflects only one spin state of light upon normal illumination without reversing the handedness of the light involves two outstanding challenges in nanophotonics. First, the intrinsic chirality, i.e., chiroptical effects for light of normal incidence, in quasi-two-dimensional lossless structures has been speculated to be prohibited^8–11^. In fact, the main requirement to achieve intrinsic chiroptical effects is a simultaneous excitation of both in-plane magnetic and electric dipole moments upon normal illumination^10,12^, where “in-plane” refers to the quasi-2D structure intended to discriminate opposite spins of light. For this condition to be fulfilled, according to a long-held notion, the structure has to be composed of complicated three-dimensional chiral elements, such as helices, or alternatively made of multilayer patterns with structural chirality^10,12^. To date, several demonstrations of plasmon-assisted intrinsic chiroptical responses in metastructures consisting of subwavelength arrays of 3D chiral shapes^10,13–15^ or multilayer patterns of mirror-symmetry-broken structures^16–21^ have been reported. Although such structures may exhibit a strong wideband chiroptical response, their fabrication is not compatible with 2D patterning techniques^22^. Top–down fabrication techniques including direct laser writing have been widely employed to create 3D chiral structures with arbitrary geometries^10^. However, such techniques are usually limited to micrometer resolution, and are therefore not suitable for nanoscale structures operating in the visible and near-infrared regions^13^. Alternative techniques such as electron-beam-induced deposition (EBID)^23^ and colloidal nanohole lithography^24^ are too complicated for manufacturing large-area plasmonic structures.

For thin, quasi-2D structures, the co-excitation of the in-plane dipole moments seems too challenging. Although in-plane electric dipoles can be readily excited, the excitation of in-plane magnetic dipole moments involves a circulation of the polarization current at the vertical cross-section of the structure, which is not straightforward to accomplish in a thin structure^11^. On the other hand, the need for an in-plane magnetic moment is eliminated with obliquely incident light, which is commonly termed extrinsic chirality^25,26^. Recently, Zhu et al.^11^ reported an experimental observation of a giant intrinsic chiroptical effect in an optically thin metasurface consisting of a periodic array of chiral gammadion-shaped meta-atoms arranged on a dielectric slab. This metasurface selectively transmits only one spin state of light while diffracting the opposite spin. The underlying physical mechanism is a selective excitation of higher-order multipoles, such as the toroidal quadrupole and magnetic octupole^27^. Since the primary radiation direction for the higher-order modes upon excitation is off-normal^27^, the transmission in the forward direction is eliminated, and the structure effectively filters out the selected helicity. However, while multipole engineering can render a structure intrinsically chiral^28,29^, it is not suitable for designing reflective chiral structures. Moreover, as will be elucidated further, polarization conversion is a main requirement of the operation of spin-preserving mirrors, and higher-order multipoles are not able to produce the desired polarization conversion for the zeroth diffraction term.

The second challenge is the requirement of handedness preservation upon normal reflection. For an ordinary mirror or any uniform dielectric interface, reversal of the helicity occurs when light reflects off the surface. Several groups have reported demonstrations of “magical mirrors” that selectively reflect circularly polarized light into the co-circular-polarization state^1,30–33^. Although diverse structures have been proposed, there is a commonality between the approaches: the proposed mirrors consist of 2D chiral arrays arranged on top of a back metallic mirror. A judicious design of such structures would enable the complete reflection of one spin state without a handedness reversal, whereas the opposite spin should be completely absorbed^1^. The operational principle of these magical mirrors is reliant on selective absorption of the light impinging on the metallic chiral pattern. However, absorption is fundamentally limited to 50% in 2D arrays^1^. It turns out that the presence of back metallic mirrors is indispensable, allowing complete absorption of the selected polarization within a round trip of propagation^1^. Despite the fact that the demonstrated mirrors can potentially lead to near-unity circular dichroism^30^, the realization of spin-preserving mirrors in monolithic planar all-dielectric structures remains a challenge.

Here, we describe the first experimental observation of maximum intrinsic chirality^34^ in a truly monolithic and lossless photonic crystal (PC) membrane with a chiral array of perforating holes. Our structure is designed and fabricated for operation in the near-infrared range. Upon normal illumination, the PC slab reflects the chosen helicity into the same state of polarization with a near-unity reflection coefficient, while the opposite spin is completely transmitted. The structure overcomes the challenges outlined above by a proper hybridization of leaky Bloch modes, leading to near-unity circular dichroism. The underlying physical mechanism is guided-mode resonance (GMR)^35^ via loosely confined TE and TM modes. The hybridization of extremely leaky (with a low radiation-quality factor) TE and TM modes entails a resonant reflection of the selected circular polarization into the co-circularly polarized state, and handedness preservation is achieved via the spatial symmetry of the perforating holes. As a result, the associated low-Q modes can effectively interact with free-space illumination, and the need for an infinitely extended structure is largely eliminated.

## Results

### Design, concepts, and device fabrication

**Fig. 1:**
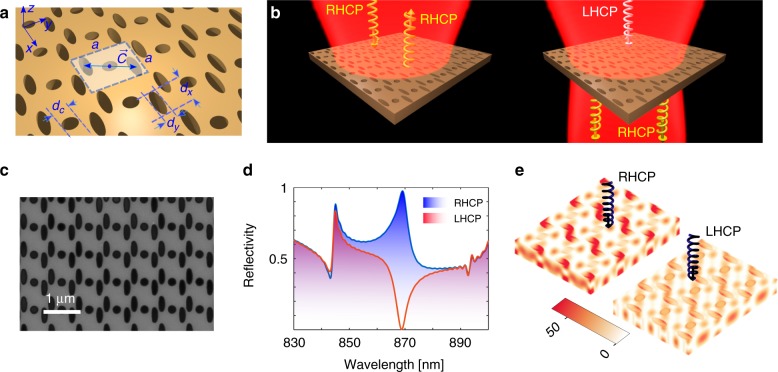
Schematics and simulation results. **a** Schematic of the chiral PC membrane and geometry definitions. The Bravais lattice is square with the lattice constant of $a = 740\;{\mathrm{nm}}$. The unit cell (shaded area) comprises a tripartite configuration of perforating holes: a circular hole at the center with a diameter of $d_c = 200\;{\mathrm{nm}}$ and two elliptical holes ($d_x = 420\;{\mathrm{nm}}$ and $d_y = 140\;{\mathrm{nm}}$) displaced by ${\pm}\!{\vec C} = {\pm}\! {\left( {150\hat x + 275\hat y} \right)}\left[ {\mathrm{{nm}}} \right]$ with respect to the center. The membrane is made from silicon nitride with a refractive index of $n\sim 2.26$ and a thickness of ${\sim}\, {309}\;{\mathrm{nm}}$. **b** Illustration of the optical response at the designed wavelength of $870\;{\mathrm{nm}}$. The structure reflects RHCP light while preserving its handedness. The opposite spin is transmitted, and its handedness is reversed. **c** SEM image of the fabricated device. **d**, **e** FDTD simulation results: **d** power reflection spectrum for the two spin states of the incident light and **e** the corresponding intensity distributions over a few unit cells. The color axis displays the normalized electric field intensity profile $\left| {E/E_0} \right|^2$, where *E* and *E*_0_ are the induced electric field magnitude upon circularly polarized illumination and the magnitude of the incident field, respectively

A schematic of the designed photonic crystal mirror is displayed in Fig. 1a. The photonic crystal membrane consists of a patterned layer of silicon nitride with a thickness of *t* ~ 309 nm that optimizes operation at the target wavelength of *λ* ~ 870 nm. The design can be adjusted for other wavelengths or dielectric materials. The Bravais lattice is square-shaped with a subwavelength lattice constant, which assures that upon free-space illumination only zeroth-order diffraction will contribute to the reflection and transmission in the far field. The photonic crystal membrane can thus be regarded as an effectively homogeneous boundary. The unit cell consists of a tripartite array of perforating holes with chiral symmetry in the *xy*-plane (see Fig. 1a). Here, chirality is achieved by engineering the detailed geometry of the unit cell, and the wavelength is adjusted by properly selecting the thickness of the slab.

Maximum electromagnetic chirality requires preservation of the light’s handedness at normal incidence, which is imposed by time reversal symmetry^34^. In essence, the symmetry of the pattern of the unit cell dictates the basic relationship between the elements of the reflection tensor. Employing Jones calculus within the circular basis, the reflection and transmission properties of the slab are then described by the elements of the 2 × 2 matrices ${\mathbf{R}} = \left[ {{\cal{R}}_{ij}} \right]$ and ${\mathbf{T}} = \left[ {{\cal{T}}_{ij}} \right]$, respectively. Henceforth, the corresponding matrix elements are subscripted by + and −, designating the right-handed and left-handed circularly polarized modes, respectively. The desired reflection properties at the designed wavelength necessitate setting ${\cal{R}}_{ + + } = 1$ (or, for the opposite enantiomer, ${\cal{R}}_{ - - } = 1$), whereas the other three elements should be vanishingly small. This assures that the PC mirror reflects only one spin state of light without reversing the handedness, whereas the opposite spin is completely transmitted. It is also worth pointing out that due to its 2D nature, the structure should exhibit opposite chirality on both sides. In stark contrast to the properties of 3D chiral objects such as a helix, where the sense of twist associated with the object is independent of the observation direction, the perceived sense of twist of a planar chiral object is reversed upon reversal of the observation direction. This, in conjunction with time reversal symmetry, entails flipping the spin of the transmitted light (see Supplementary Material). Therefore, in an ideal scenario, the only nonzero element of the corresponding transmission matrix at the target wavelength is $\left| {{\cal{T}}_{ + - }} \right| = 1$ ($\left| {{\cal{T}}_{ - + }} \right| = 1$ for the opposite enantiomer). Figure 1b schematically illustrates the expected optical response of the PC slab at the designed wavelength.

The required structure of the reflection and transmission tensors defined above has several consequences and poses further limitations on the spatial symmetries of the perforating holes. As detailed in the Supplementary Material, only the onefold *C*_1_ and twofold *C*_2_ symmetry groups accompanied with broken mirror symmetry in the *xy*-plane will fundamentally allow preservation of the helicity (spin) upon reflection. In other words, the necessary condition to realize such spin-preserving mirrors is to simultaneously break the *n*-fold rotational symmetry (for *n* > 2) and any in-plane mirror symmetries^9,30^. To also reduce the sensitivity of the structure with respect to the angle of incidence, we elected to design a unit cell with twofold rotational symmetry (see Fig. 1a). The dimensions were initially selected using band diagram analysis, and finely adjusted for a maximum extinction ratio and near-perfect reflection through a brute force-optimization technique. The optimization is constrained with the fabrication limitations, including the bridge sizes between adjacent holes and the smallest curvatures to be etched. An SEM image of the fabricated photonic crystal membrane is shown in Fig. 1c.

The structure was simulated with the finite difference time-domain (FDTD) method using a commercial solver (Lumerical Inc.). The power reflectivity of the slab for normal incidence of the RHCP and LHCP light and the corresponding field distributions are shown in Fig. 1d, e, respectively. The simulation results promise >97% reflection for one spin state of light at the target wavelength, whereas the opposite spin state is almost completely transmitted. The extinction ratio can reach up to 1000, which is unprecedented among the relevant works^30^. This giant intrinsic chirality originates from the guided-mode resonance (GMR) mediated by two extremely leaky Bloch modes across the band edges of the PC slab. Due to bi-modal interference, the intensity profiles at the cross-section of the photonic crystal slab (shown in Fig. 1e) are asymmetric and symmetric for the reflected and transmitted helicities, respectively.

**Fig. 2:**
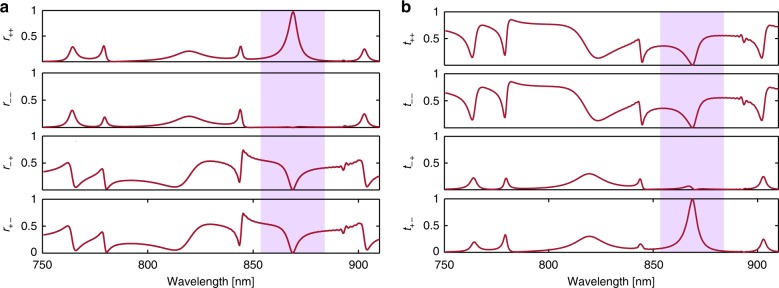
Elements of the Jones matrices. Simulation results for **a** the power reflection and **b** transmission coefficients. The subscripts + and − denote the RHCP and LHCP modes, respectively. The bandwidth of the maximum chirality is shaded

The simulated polarization-resolved reflection and transmission coefficients are displayed in Fig. 2. The power reflection and transmission coefficients, denoted by $r_{ij}$ and $t_{ij}$, respectively, are related to the elements of the Jones matrices as $r_{ij} = \left| {{\cal{R}}_{ij}} \right|^2$ and $t_{ij} = \left| {{\cal{T}}_{ij}} \right|^2$. The results confirm that over the operational frequency band (the shaded region in Fig. 2), the photonic crystal is maximally chiral^34^; at the target wavelength, the photonic crystal selectively reflects the light and retains its handedness and, in compliance with the symmetry constraints, transmits the opposite spin while flipping the helicity.

This realization of maximum intrinsic chirality in a monolithic structure is exceedingly surprising. As noted earlier, the origin of chiroptical effects can be traced back to the simultaneous excitation of effective in-plane magnetic and electric moments induced within the building blocks of the structure^11,36^. Using the dipolar approximation, the optical response of the structure can be described on the basis of the net electric dipole per unit cell, i.e., ${\mathbf{p}} = {\frac{1}{i\omega}\int\!\int\!\int {\mathbf{J}}{\mathrm{d}}v}$, and the net magnetic dipole moment calculated as ${\mathbf{m}} = \frac{1}{{2c}}{\int\int\int} {{\mathbf{r}} \times {\mathbf{J}}{\mathrm{d}}v}$, where **J**, ω, and *c* are the polarization current, frequency, and speed of light in vacuum, respectively. Since both the electric and magnetic dipole modes radiate primarily along the directions normal to their axis, their cooperative action requires a co-planar and co-linear excitation of the moments so that ${\mathbf{p}}_\parallel \cdot {\mathbf{m}}_\parallel\; \ne\; 0$, where ${\mathbf{p}}_\parallel$ and ${\mathbf{m}}_\parallel$ refer to the components of the dipoles tangential to the plane of the slab^36^. For this to occur, the slab should be thick enough so the polarization/displacement current can be circulated within the vertical cross-section of the structure. In contrast to conventional wisdom, however, giant circular dichroism with maximum chirality occurs in a PC slab whose thickness is much less than the target operation wavelength.

**Fig. 3:**
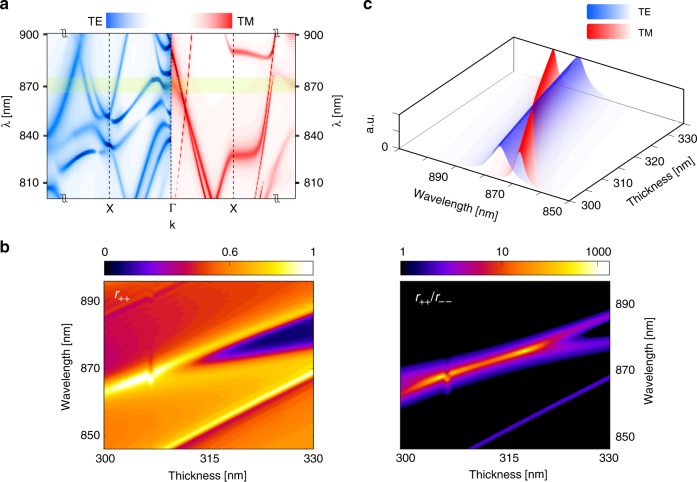
Band diagram and mode hybridization plots. Two low-Q TE and TM modes are hybridized by varying the thickness of the slab. For the thickness of $t\sim 309\;{\mathrm{nm}}$, the modes are properly hybridized, guaranteeing the co-excitation of in-plane magnetic and electric dipole moments at the target wavelength of $870\;{\mathrm{nm}}$. **a** Relevant part of the band diagram for the TE (blue) and TM (red) modes. The color axis displays the spectral function obtained from the time-domain simulations. High-Q Bloch modes are brighter as they exhibit stronger resonance. The displayed part of the band is within the radiation continuum (above the light cone), and the yellow-shaded region is the operation band of the mirror. **b** The total reflectively of the chosen helicity, $r_{ + + }$, is shown on the left, and the extinction ratio, $r_{ + + }/r_{ - - }$, is shown on the right. The hybridization causes extreme chirality manifested in a large extinction ratio. **c** Spectral distribution of the TE and TM modes. The distributions are normalized, and thus display the center wavelength and linewidth associated with each Bloch mode. The modes are extremely leaky and thus have low radiation quality factors, which in turn leads to GMR

The key to understanding the operational principles of our chiral PC mirror is guided-mode resonance. Here, maximum intrinsic chirality is achieved by engineering low-Q TE-like and TM-like modes within the radiation continuum. Since the Bloch modes across the band edges of the photonic crystal structure have a low lateral expansion velocity, the modes just radiate back into free space and thus effectively act similarly to the Mie and Fabry–Pérot resonant modes in dielectric metasurfaces and supercavities^37^. The details of the geometry of the unit cell allow us to adjust the radiation quality factor associated with each virtual resonant mode so that the modes become extremely leaky. Leaky modes provide an efficient way to channel light from within the slab to the external environment via radiation, and at the same time, the judicious design of the structure leads to the formation of TM modes that extend well outside of the slab. Thanks to the latter, desired magnetic moments can be excited. The TE-like modes can effectively generate the desired in-plane electric dipole moment, and the in-plane magnetic dipole is generated by the TM-like modes within the radiation continuum. If the slab dimensions are properly selected, the TE-like and TM-like modes acquire degenerate resonance frequencies, which allows their hybridization. This in turn gives rise to strong intrinsic chirality. Since the radiation channels associated with the TE-like and TM-like modes are not fully orthogonal, via-continuum coupling occurs, leading to a slight removal of the degeneracy^37,38^. The relevant part of the band diagram around the band edge i.e., k = 0 (denoted by Γ) is displayed in Fig. 3a. The band diagram is obtained by a FDTD simulation with a Bloch boundary condition applied to a single unit cell. The simulation involves placing random dipole sources in the unit cell so that all possible modes are excited and recording the field in the time domain. High-Q modes are less leaky and therefore have longer lifetimes upon excitation. The color axis displays the spectral function obtained from the FDTD simulation; the modes with less radiation leakage exhibit stronger resonance and are thus brighter. Within the yellow-shaded frequency band, the desired low-Q TE and TM modes meet each other at the band edge, which enables their hybridization.

The anomalous reflection of photonic crystals occurs due to the interference of leaky Bloch modes and the continuum of unbounded modes and thus exhibits a Fano-shape spectral profile^39^. The electromagnetic dipole moments in the helical basis (which are commonly called σ-dipoles^40^) consist of parallel magnetic and electric dipole moments of equal amplitudes that are phase shifted by ±π/2. The presence of resonance based on σ-dipole gives rise to the chiroptical effects of interest. At the design wavelength, the reflection from our PC mirror is generated by a resonant coupling of circularly polarized light to σ-dipoles, which are essentially produced by the co-excitation of TE-like and TM-like modes and through the background reflection.

To reveal how mode hybridization leads to chirality, we performed parameter tuning to decouple the modes. The thickness of the slab was varied to observe the variations in the reflectively of the chosen helicity and the extinction ratio as the figures of merit. Figure 3b shows the investigated crossing region with the reflectivity results from finely sampled simulations. The spectral distributions of the associated TE and TM modes, calculated using the FEM method (ANSYS HFSS Inc.), are shown in Fig. 3c (the electric field profile for each mode is shown in the Supplementary Material, Fig. S2). The distributions are normalized and exhibit the resonance wavelength and linewidth associated with each Bloch mode. To confirm that mode crossing occurs, we applied a mode tracing scheme. As expected, the TE-TM-mode crossing occurs around the predicted thickness.

**Fig. 4:**
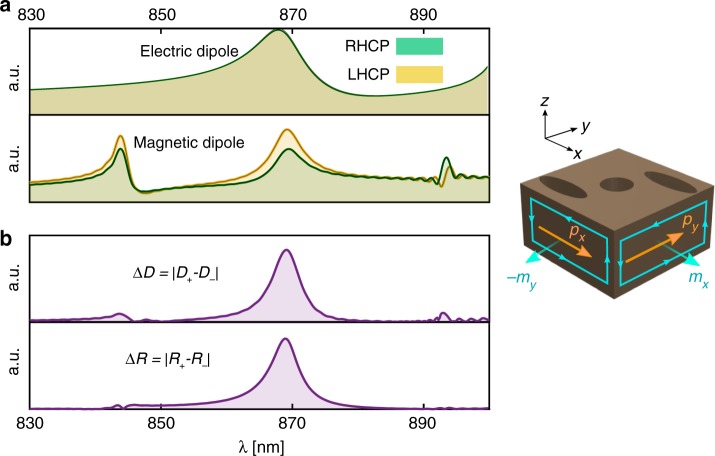
Spectral variations of the induced dipole moments. **a** Dipole moments for RHCP and LHCP incidence (the green- and yellow-shaded curves, respectively). The in-plane electric dipole $\left| {{\mathbf{p}}_ \pm } \right|$ and the magnetic $\left| {{\mathbf{m}}_ \pm } \right|$ are displayed in the upper and lower plots, respectively. Here, the subscripts “+” and “−” denote RHCP and LHCP light, respectively. **b** Inner product of the in-plane electric and magnetic dipoles, i.e., $D_ \pm = \left| {{\mathbf{p}}_ \pm \cdot {\mathbf{m}}_ \pm } \right|$ and the reflectivity contrast $\Delta R = \left| {R_ + - R_ - } \right|$. The reflectivity contrast $\Delta R$ follows $\Delta D = \left| {D_ + - D_ - } \right|$

To more closely study the dipolar interpretation of our structure’s predicted behavior, we studied the impact of dipole interactions. We calculated the in-plane components of the induced electric dipole **p** and the magnetic dipole **m** per unit cell for incident light of both circular polarizations. The toroidal dipoles and the higher-order multipoles were found to have negligible contributions. Figure 4a displays the spectral distribution of the induced dipole moments. The induced electric dipoles are identically excited for opposite helicities, whereas the strengths of the magnetic dipoles are different over the chiral bands. Around the design wavelength, where the modes are properly hybridized, the inner products of the resultant dipoles are distinctly different for the opposite helicities. Intriguingly, the circular dichroism upon reflection follows the magneto-electrical dipole interactions (see Fig. 4b).

The origin of the difference in the induced magnetic dipoles shown in Fig. 4a can be explained based on symmetry considerations. The induced polarization currents for circularly polarized incident fields can be naturally decomposed into a chiral component and an achiral component, namely, **J**_**c**_ and **J**_**a**_, respectively. The chiral component **J**_**c**_ is induced differently for the opposite helicities and has in-plane chiral symmetry in its distribution. In addition, owing to the twofold rotational symmetry, **J**_**c**_ has a definite parity under in-plane space inversion. Specifically, it is observed that the chiral polarization vector is an odd-parity vector field i.e., ${\mathcal{P}}_{xy}${**J**_c_} = −**J**_**c**_, where ${\mathcal{P}}_{xy}$ is the in-plane parity operator. Since the electric dipole moment is obtained by a direct integration of the polarization current over the unit cell, the net electric dipole moment loses its in-plane chiral features. Therefore, the electric dipole moments are equally excited for both helicities of the incident light. In contrast, as the net magnetic dipole moment is calculated by a direct integration of **r** × **J** and ${\mathcal{P}}_{xy}${**r** × **J**_**c**_} = + **r** × **J**_**c**_, the chiral polarization components should have a nonvanishing contribution in the magnetic dipole moment, which in turn leads to the excitation contrast observed in Fig. 4a.

The magneto-electric dipole excitation described above is fundamentally different from the operational mechanism of lossless planar dielectric metasurfaces made of high refractive-index nanopillars. Analogous to high-contrast gratings that essentially operate in a dual-mode regime^41^, such all-dielectric metasurfaces support multiple-guided modes, and the formation of supermodes in a symmetry-broken geometry can result in asymmetric transmission for orthogonal polarizations. Therefore, the nanopillars need to be sufficiently tall to accommodate internal multimode propagation. It has been recently demonstrated that a judicious design of nanopillars with in-plane geometrical chirality can potentially result in different couplings of opposite helicities to the waveguide-array modes, which in turn leads to a differential response for left- and right-handed circularly polarized light^42^. Ye et al.^43^ showed that multimode interference can also arise in metasurfaces made of low-loss metallic nanostructures with finite thickness. Consequently, properly designed metallic nanoposts can support surface plasmon modes whose different interference schemes for the opposite helicities yield a giant chiroptical effect^43^.

### Experimental results

The silicon nitride layer for our structures was grown on a silicon wafer using low-pressure chemical vapor deposition (LPCVD), producing a film with a refractive index of 2.26 at the designed wavelength. The structures were fabricated through soft-mask electron-beam lithography followed by plasma etching and a KOH undercut. This process results in a truly free-standing photonic crystal membrane, and the undercut area is sufficiently deep so that the impact of silicon substrate can be safely disregarded. A scanning electron microscopy (SEM) image of the fabricated device is displayed in Fig. 1c.

The experimental characterization of the photonic crystal mirror was carried out by free-space illumination with the beam of a supercontinuum white-light laser. The beam was focused onto the PC sample through a low-numerical-aperture objective lens to assure that the wavefront of the excitation remains similar to a plane wave. To obtain the reflection spectrum, we collected the reflected light into a spectrometer via a single-mode fibre. The focused beam at the sample was approximated by a Gaussian profile; the corresponding beam waist at the sample was estimated to be $w_0 \approx 18\;\upmu {\mathrm{m}}$. To eliminate artefacts originating from unwanted rays, the reflected beam passed through a confocal reflectometry setup with appropriate polarimetric arrangements. The setup was designed to monitor the four components of the power reflection matrix in the circular basis: $r_{ + + }$, $r_{ - - }$, $r_{ + - }$, and $r_{ - + }$. The confocal configuration is necessary to compensate for the long depth of field associated with the loosely focused beam so that the rays reflecting off the undercut area are largely avoided. To further reduce the interference of the rays reflecting from the thick silicon layer underneath the PC membrane, the undercut region has a V-shaped cross-section in the silicon substrate; thus, the reflections from the undercut region are mainly off-normal. Additional details of the optical setup can be found in the Supplementary Material (see Supplementary Figs. S6 and S7).

**Fig. 5:**
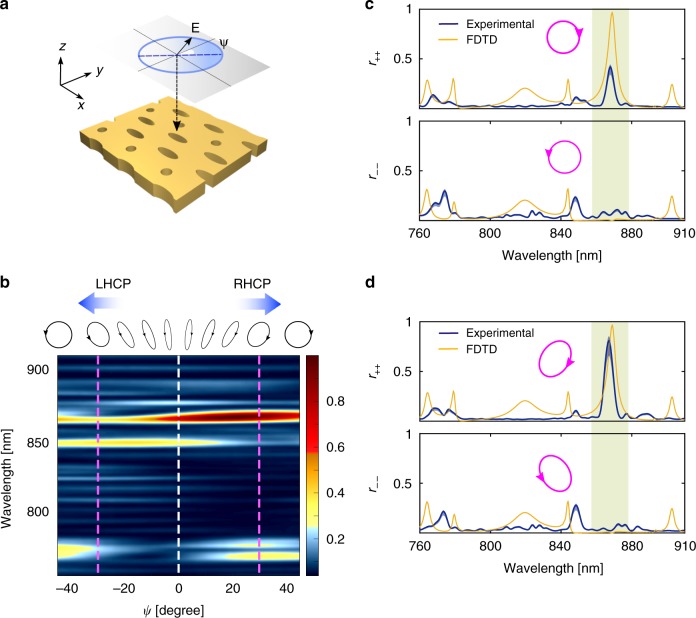
Experimental results. **a** Polarization ellipse of the incident wave generated by the quarter-wave plate placed before the focusing objective lens. The major axis is tilted by the angle $\psi$ with respect to the polarization of the input beam. The axial ratio is $\tan \psi$. For $\psi = \pm\!45^\circ$, the incident light is circularly polarized. **b** A color plot of the measured normalized reflection spectrum for different elliptically polarized beams. Around the design wavelength, the eigenpolarizations that exhibit maximum reflectivity and chirality are elliptical. **c** Reflectivity of circularly polarized incident light-to-light with the same helicity. Due to fabrication imperfections, the measured reflectively is relatively poor (only ${\sim} {50\%}$). **d** Reflection spectrum of elliptically polarized light with $\psi \sim \pm\! 30^\circ$ (for right-handed and left-handed elliptically polarized light) to the same polarization. The extinction ratio at a wavelength of ${\sim} 868\;{\mathrm{nm}}$ is $\frac{{r_{ + + }}}{{r_{ - - }}}\sim 33$

Due to a slight astigmatism of the electron beam during the lithography process and imperfect etching, the fabricated samples exhibit some anisotropy that leads to a modest performance degradation of the PC mirror. Specifically, it was observed that the eigenpolarizations are not purely circular. To experimentally explore this effect, we carried out polarization-dependent reflectometry. The polarization of the incident light is adjusted by means of a broadband quarter-wave plate placed before the objective lens. This setup allows us to explore a wide range of elliptical polarizations for the incident light. The total reflectivity of the PC mirror for different states of polarization is shown in Fig. 5b. Note that all of the reflectivity measurements are calibrated based on the known reflectivity of the unpatterned silicon nitride on a silicon substrate. It is observed that around the target wavelength of ${\sim} 870\;{\mathrm{nm}}$, the sample exhibits extreme chirality. However, near-unity reflection occurs for right-handed elliptically polarized light with an axial ratio of $AR = \tan 32^\circ \approx 2:3$ (the dark red spot in Fig. 5b). We emphasize that due to the resonant nature of the chiral reflection mechanism, the sensitivity to structural deformations is expected to be pronounced. However, refinements to our fabrication procedure should allow us to make the eigenmodes purely circular. The observed deviation from circular to elliptical eigenpolarizations indicates a cross-coupling between the opposite spins. To inversely reconstruct the actual fabricated device, we carried out a diagnostic analysis based on the adjoint shape optimization technique^44^. The analysis revealed that due to the imperfect etching, the walls of the photonic crystal holes are not perfectly vertical, so the diameters of the holes at the bottom and top surfaces are slightly different. This small imperfection was observed in a zoomed SEM image of the device as two concentric boundaries appearing around the individual holes (see Supplementary Material, Fig. S6). In agreement with intuition, mirror symmetry breaking in the *z*-direction causes an additional cross-coupling between the associated TE-like and TM-like modes, which in turn leads to the presence of the off-diagonal elements ${\cal{R}}_{ + - }$ and ${\cal{R}}_{ - + }$ in the reflection matrix.

The measured spin-preserving components of the reflectivity tensor for circular and elliptical polarizations are shown in Fig. 5c, d, respectively. The off-diagonal elements are less pronounced within the chiral band and are presented in the Supplementary Material (see Supplementary Fig. S5). There are some discrepancies between the simulations and experimental results, including a few nanometers wavelength offset between the dominant features of the simulated and experimentally observed spectra. However, when overlaying the simulations and experimental results, we observe very good agreement overall, and the slight differences most likely arise from fabrication imperfections. The experimental results confirm the selective reflection of the incident light for two elliptically polarized eigenmodes with opposite helicity with an extinction ratio up to $r_{ + + }/r_{ - - }\,\, {\gtrsim}\!\,\,30$. Such extreme chirality is unprecedented compared to the previously reported experimental results. The blue shaded reliability curves account for uncertainties in the calibration of the reflectivity measurement results, including a slight loss of coupling to the single-mode fibre when the illuminated spot is moved to the unpatterned area. It is also worth pointing out that the slightly smaller measured reflectivity compared with the simulation results can be partly attributed to the finite size of the focused beam at the sample. As is further evidenced by the plane-wave expansion analysis presented in the Supplementary Material, the Gaussian beam used in our experiment contains obliquely incident plane waves that have significantly lower reflection coefficients (see Supplementary Material, Fig. S8); thus, even in an ideal scenario, the reflectivity of such a Gaussian beam cannot exceed ${\sim} 85\% .$

**Fig. 6:**
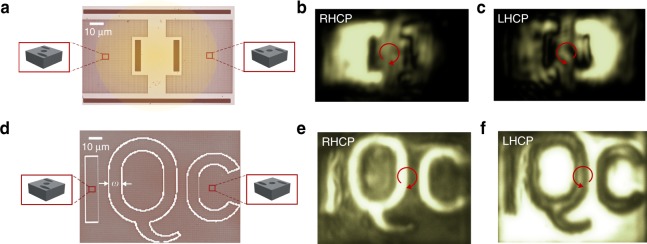
Reflection of the opposite enantiomers upon circularly polarized illumination. **a**–**c** Imaging using a focused and spatially coherent laser source. **a** Optical microscope image of the fabricated pattern. The left “C” structure reflects RHCP light, while the “mirror C” structure on the right only reflects LHCP light. Each C is $50\;\upmu {\mathrm{m}} \times 50\;\upmu {\mathrm{m}}$. The yellow-shaded circle shows the region illuminated by the coherent laser beam (**b, c**) reflection of CP light to its co-circular polarization. **d**–**f** Imaging using a laser beam with a scrambled wavefront. The interior and exterior of the letters (IQC) are made of photonic crystals with opposite chiral patterns. **d** Optical microscope image of the fabricated IQC pattern. The letters are ~$10\;\upmu {\mathrm{m}}$ wide; i.e., $w\sim 10\;\upmu {\mathrm{m}}$. **e** ${\mathrm{RHCP}} \to {\mathrm{RHCP}}$ and **f** ${\mathrm{LHCP}} \to {\mathrm{LHCP}}$ reflection imaging. In both cases, extreme chirality occurs at a wavelength of ${\sim} 824\;{\rm{nm}}$, which is the wavelength of the laser source

To demonstrate the robust performance of the chiral mirrors, we performed two experiments involving polarization-resolved imaging. In the first experiment, two C-shaped photonic crystal membranes with opposite chiral patterns (two enantiomeric configurations) were fabricated. We identified the chiral operational band of the PC sample through polarization-resolved spectroscopy. An optical microscope image of the fabricated sample is displayed in Fig. 6a. The pattern was then illuminated by a monochromatic and spatially coherent laser beam from a tunable continuous-wave Ti:Sapph laser at 824 nm, the wavelength at which the PC sample exhibits extreme chirality. Polarization-resolved images are shown in Fig. 6b, c. The images are captured after a circular-polarization filter collecting only the spin-preserving (co-circularly polarized) reflection; thus, artefacts originating from the background are largely suppressed. Under purely circularly polarized illumination, only one of the photonic crystal structures appears bright, with the fringing in the image arising from the high level of spatial coherence of the illumination in this case.

We also created a pattern of letters (IQC) as shown in Fig. 6d. The interior and exterior of the letters are made of photonic crystal structures with opposite enantiomeric patterns. Following the experimental procedure outlined above, we again performed polarization-resolved imaging. This time, however, we illuminated the pattern with a monochromatic but spatially incoherent laser beam. To suppress the beam’s spatial coherence but not its temporal coherence, the laser beam was first focused onto rotating ground glass diffusers and collimated again^45^. As can be seen in Fig. 6e, f, the letters appear bright with clear contrast with respect to the background under RHCP illumination, while for LHCP illumination, the exterior region appears bright and the letters are dark. Intriguingly, the contrast between complementary domains is relatively high, even around the sharp edges. Since the letters are ~10 μm wide, this result demonstrates that strong chirality can be achieved even for photonic crystal structures that are periodic over small scale areas. This observation is consistent with the results obtained from full-wave simulations of a number of finite-size photonic crystal slabs presented in Supplementary Material (see Supplementary Fig. S9).

## Discussion

Compared to state-of-the-art chiral metasurfaces, including those made of complicated 3D chiral shapes^13,22,25^, our PC mirror exhibits a remarkably large chirality, despite its structural simplicity. The reflectivity of the chosen helicity to the same state of polarization can, in the ideal limit, reach up to ~100%, and we observed ~80% reflectivity with an extinction ratio above $30\!:\!1$ in our fabricated devices. In contrast, the majority of the experimental demonstrations of chiroptical effects in the visible and near-infrared range have reported circular dichroism in the transmission^11,14–18,20–22,29,46^. Among these demonstrations, the most notable examples included a demonstration of a chiral metasurface by Zhu et al.^11^, which achieved spin filtering with an extinction ratio of ~9:1 while almost ~90% of the light with the selected helicity was transmitted, and a planarized chiral structure made of multiple layers of twisted metamaterials by Zhao et al.^16^. The latter structure exhibited an almost ~30% transmission difference (and an extinction ratio of ~3:1) for the opposite helicities. However, it appears that the performance can be further improved by using a larger number of stacking layers. Most recently, a double-layer plasmonic chiral structure was proposed by Chen et al.^20^. Although this structure enables filtering opposite spins with a sufficiently large extinction ratio, the maximum transmission of the chosen helicity in the chiral band remains below ~8%. The reported experimental demonstrations of chiral mirrors, including spin-preserving mirrors, have taken advantage of a supporting back metallic/dielectric reflector in conjunction with a chiral structure that eventually boosts the reflection of the chosen helicity^19,31,32^. Our PC chiral mirror, however, genuinely combines both functionalities, i.e., spin selectivity and reflectivity, through the Bloch modes within the radiation continuum. The most notable experimental demonstration of a nanoscale chiral spin-preserving mirror was presented by Kang et al.^31^ and Ye et al.^43^. The reported experimental results^31^ indicated reflectivities of ~80% and ~20% for the intended and rejected helicities, respectively (corresponding to a reflectivity contrast of ~60% and an extinction ratio of ~4:1). Our PC mirror outperforms most of the chiral mirrors realized to date in most categories, although its operational mechanism introduces some sensitivity to the angle of incidence (see Supplementary Material, Fig. S10) and its resonant nature gives rise to a relatively narrow operation band and a certain susceptibility to fabrication imperfections. However, the structure can be further optimized for broadband operation^6^, which remains the next step for further developments. At the same time, there are a number of applications, such as gas lasers, for which a narrow operation band is not really a liability and can even be viewed as an asset for improved coherent operations. It is worth pointing out that the maximum angle of view is limited by the region of the k-space, where the group velocities of the associated Bloch modes remain negligibly small. According to Fig. 3a, the dispersion of the TE and TM Bloch modes (within the shaded area) is fairly flat over only a limited region within the k-space. Band flattening techniques^47^ may thus be employed to design a chiral PC mirror that is less sensitive to the angle of incidence.

In summary, we have designed, fabricated and experimentally demonstrated an intrinsically chiral photonic crystal mirror that, upon normal illumination, selectively reflects circularly polarized light without reversing its handedness. Guided-mode resonance resulting from the interplay of TE and TM modes across the band edge leads to enormously strong chirality with near-unity reflectivity contrast. Although the structure exhibits certain sensitivity to the angle of incidence, the extreme chirality makes this photonic crystal mirror a compelling device compared to the devices of seminal works in this area of nanophotonics.

## Materials and methods

### Sample fabrication

The photonic crystal membranes are fabricated from silicon nitride, a material that provides low absorption in the near-IR region for which the mirrors are designed to be highly reflective. The silicon nitride is grown on a 4-inch silicon wafer by low-pressure chemical vapor deposition (LPCVD) to produce a film with a refractive index of 2.26 at 850 nm. The wafer is diced into 8 × 8 mm chips using a thick (~1 μm) protective layer of PMMA.

Since the exact thickness of the silicon nitride is not always consistent and can have spatial variance over the wafer, we grow the silicon nitride to be larger than the desired membrane thickness. We then remove the protective PMMA layer with Remover PG in an 80 °C bath with sonication and use reactive ion etching (RIE) to etch the silicon nitride on a given chip to the correct thickness. The plasma etching procedure employs a 130/80 sccm mixture of C_4_F_8_/SF_6_ at 10 mTorr, with an ICP RF power of 1000 W, a platen RF power of 30 W, and a platen temperature of 15 °C. Before running the etching process, we first condition the chamber with these etching conditions for 45 min. The silicon nitride etching rates before each run are determined by running the process for 1 min on a test chip and then measuring the silicon nitride thickness before and after etching using filmetrics. Typical etch rates are 15–20 nm/min.

The fabrication of the PC pattern is performed by electron-beam lithography in combination with dry and wet etching processes. We begin by spinning ZEP520A (Zeon Chemicals) positive resist to a thickness of ~700 nm using a spin speed of 1500 rpm for 60 s at a ramp rate of 3000 rpm/min and baking for 2 min at 180 °C. The resist is developed using amyl acetate for 90 s after being exposed to the PC pattern by e-beam lithography at 100 keV. The transfer of the pattern from the e-beam resist to the silicon nitride is achieved again by RIE using the previously described silicon nitride procedure. The sample is etched to 125% of the thickness to ensure complete penetration through the film, leaving perpendicular sidewalls.

To reduce the back reflections from the silicon surface, we introduce a silicon undercut using 45% KOH solution at 80 °C, which allows for the patterned regions of the silicon nitride to become free-standing films. Typically, the silicon wet etch is performed for ~1 h, followed by immersing the sample in two beakers of deionized (DI) water for 5 min each to neutralize the KOH. These neutralizing DI baths are also held at 80 °C to prevent the formation of crystals on the surface of the sample. The samples are then transferred into two solutions of IPA for at least 5 min each to remove the DI water in the features of the pattern. This provides a surrounding solvent with a much lower surface tension, reducing the risk of breaking or cracking the delicate free-standing PC patterned films when finally dried with N_2_ gas.

### Measurement procedure

The microscope setup (shown in Supplementary Material, Fig. S3) employs a 5× objective with a numerical aperture of NA = 0.1 and a lens system consisting of two confocal lenses (Thorlabs AC254-200-B and AC254-150-B) with focal distances of 20 cm and 15 cm, respectively. The diameter of the focused beam at the PC sample was measured by a sharp blade mounted on a high-resolution translation stage. We observed a beam waist of $w_0 = 18\;\upmu {\mathrm{m}}$ with no noticeable chromatic aberration between the wavelength range of 600 nm to 920 nm. To ensure that the incident beam is precisely normal to the PC mirror, we initially align the beam without the objective so that the reflected beam is co-linear with the incident beam. The incoming beam is vertically polarized and passes through a nonpolarizing beam splitter (Thorlabs BS014). To extract the four components of the Jones matrix in the circular basis, we use a zero-order achromatic λ/4 plate (Thorlabs AQWP05M-980) immediately before the objective and after the nonpolarizing beam splitter. This setup ensures that the wavefront is not distorted and eliminates the need for a further calibration of the wave plate. For $\theta = {\pm}\! 45^\circ$ (where *θ* is the angle of the fast axis of the quarter-wave plate with respect to the vertical direction), the PC mirror is illuminated by RHCP and LHCP light. After a round trip of propagation, the diagonal elements of the Jones matrix are coupled to the vertical polarization, and the off-diagonal elements are coupled into the horizontal polarization at the output. We use a zero-order achromatic *λ*/2 plate (Thorlabs AHWP05M-980) together with a polarizer to selectively couple the reflected beam into a single-mode fibre that is connected to a spectrometer.

## Supplementary information

Supplementary Information
