# Supplementary material for: Spin-preserving chiral photonic crystal mirror
Source: Light Sci Appl. 2020 Feb 20;9:23. doi: 10.1038/s41377-020-0256-5 (PMC7033220; doi:10.1038/s41377-020-0256-5)
Supplement: Supplementary file 1 — Supplementary Information [file 41377_2020_256_MOESM1_ESM.pdf]

Supplemental Material for

Spin-Preserving Chiral Photonic Crystal Mirror

Behrooz Semnani[1,2,*] Jeremy Flannery[1,3], Rubayet Al Maruf[1,2], and Michal Bajcsy[1,2,3]

[1]*Institute for Quantum Computing (IQC), University of Waterloo, Waterloo, ON, Canada*

[2]*Department of Electrical & Computer Engineering, University of Waterloo, Waterloo, ON, Canada*

[3]*Department of Physics & Astronomy, University of Waterloo, Waterloo, ON, Canada*

E-mail: *bsemnani@uwaterloo.ca

Symmetry Considerations

Time Reversal Symmetry

We assume that a 2D chiral structure behaves as a perfect spin-preserving mirror so that upon normal illumination of a circularly polarized light, the structure completely reflects a chosen helicity whereas the opposite spin is transmitted. Without loss of generality, we assume that the structure reflects the right handed circularly polarized (RHCP) lights impinging onto the top surface of the structure, to the same state of polarization i.e. $\mathcal{R}_{++} = 1$ whereas $\mathcal{R}_{--} = \mathcal{R}_{+-} = \mathcal{R}_{-+} = 0$ (on the top side). Here we show that for the nonreflective helicity, the time-reversal symmetry results in flipping the spin in transmission i.e. $|\mathcal{T}_{+-}| = 1$. The analysis presented here stands on two symmetry considerations; first, since the structure is assumed to be lossless, upon applying the time reversal symmetry operator, the reflection and transmission tensors should consistently describe the essential physics. Second, due to the 2D nature of the structure, the sense of twist associated with the chiral pattern is reversed upon reversal of the observation direction. In other words, if the structure reflects the RHCP lights that are illuminated from the region above the mirror, it should reflect LHCP light when the light is illuminated from the region below the 2D structure. This effect is exclusive to 2D chiral shapes with no 3D chiral features.

For the sake of mathematical compactness we use the Dirac notations to specify the state of incident and scattered lights as $|\alpha, \beta, \gamma\rangle$ where $\alpha = \pm\hat{\mathbf{k}}$ specifies the direction of the propagation (see Fig. S1A), $\beta = R, L$ denotes the handedness and $\gamma = top, bottom$ denotes the upper or lower half-spaces. Upon applying time reversal operator $\hat{T}$ on a plane wave with a given helicity, its propagation direction is reversed. However the handedness remains unchanged and obviously it stays within the same half space. Therefore one can write $\hat{T}A|\alpha, \beta, \gamma\rangle = A^*|-\alpha, \beta, \gamma\rangle$ where the complex conjugate accounts for the anti-unitary nature of the time reversal operator.

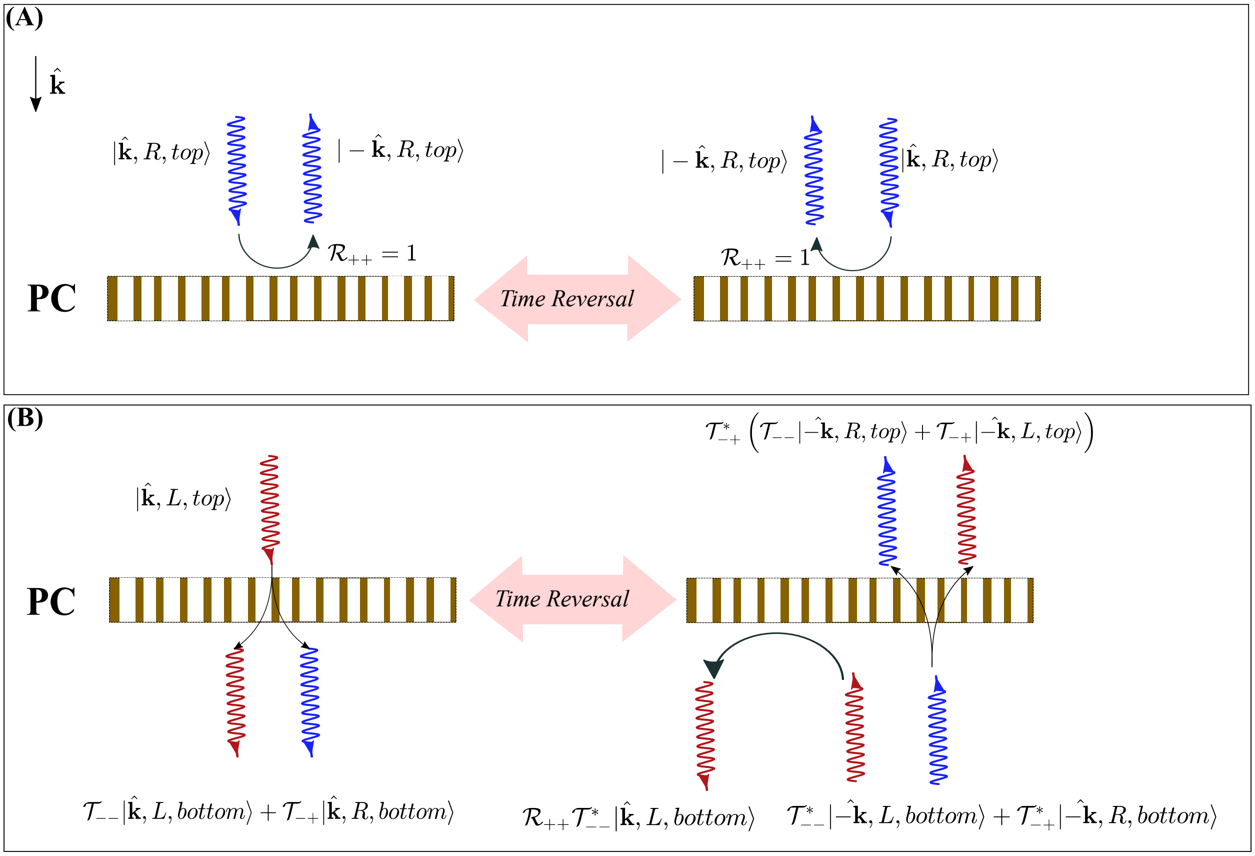

Figure S1: **Time reversal symmetry:** the right schematics display the time reversed version of the interactions. The structure completely reflects the RHCP light being illuminated from the upper half-space. However, if light is illuminated from the bottom it should be LHCP to be completed reflected. Upon applying the time reversal operator two times , one can conclude that $|\mathcal{T}_{-+}| = 1$.

Fig. S1 displays the optical response of the PC mirror for RHCP and LHCP illuminations and the associated time reversed version of the interactions are shown on the right panels. In order to have physically consistent processes, one should be able to bilaterally link the time reversed pictures. Taking $\mathcal{R}_{++} = 1$ as the initial assumption, the right and left panels are linked if and only if

$$\mathcal{R}_{++}\mathcal{T}^*_{--} * |\hat{\mathbf{k}}, L, bottom\rangle = 0 \tag{1}$$

$$\mathcal{T}^*_{-+}\mathcal{T}_{--}|-\hat{\mathbf{k}}, R, top\rangle = 0 \tag{2}$$

$$|\mathcal{T}^*_{-+}|^2\,|-\hat{\mathbf{k}}, L, top\rangle = \hat{T}|\hat{\mathbf{k}}, L, top\rangle \tag{3}$$

which necessitates setting $\mathcal{T}_{--} = 0$ and $|\mathcal{T}_{-+}| = 1$. Similar arguments can be applied to show that $\mathcal{T}_{++} = \mathcal{T}_{+-} = 0$.

The coefficients $\mathcal{T}_{ij}$s have been obtained for the lower half-space. If the structure is exposed to circularly polarized plane waves illuminated from the upper half-space, the chirality is reversed and therefore the only nonzero element of the transmission matrix is $|\mathcal{T}_{+-}| = 1$

Spatial Group Symmetries

Spin preservation upon reflection poses further limitations on the spatial group symmetries of the structure. For simplicity we can take the aforementioned assumptions (i.e $\mathcal{R}_{++} = 1$ while the other elements are vanishingly small). We also assume that 2D chiral mirror sits is placed on the xy-plane. By applying the rotation operator $\hat{R}_z(\varphi)$ on the coordinate system (moving to a new coordinate $x'y'z'$), the circularly polarized basis are transformed as

$$\hat{x} + i\hat{y} = e^{i\varphi}(\hat{x}' + i\hat{y}') \tag{4}$$

$$\hat{x} - i\hat{y} = e^{-i\varphi}(\hat{x}' - i\hat{y}') \tag{5}$$

Noting that the direction of the propagation for the reflected helicity is reversed, the spin preserving terms couple $\hat{x} + i\hat{y}$ to $\hat{x} - i\hat{y}$ and thus by rotating the coordinate system, the reflection matrix is transformed as

$$\mathcal{R}'_{++} = \exp(i2\varphi)\mathcal{R}_{++}$$
$$\mathcal{R}'_{--} = \exp(-i2\varphi)\mathcal{R}_{--}$$
$$\mathcal{R}'_{-+} = \mathcal{R}_{-+}$$
$$\mathcal{R}'_{+-} = \mathcal{R}_{+-}$$

However, if the chiral mirror has an n-fold rotational symmetry so that upon rotating the structure around the z-axis by the angle of $2\pi/n$ the structure remains unchanged, then the above condition is violated except for $n = 1, 2$. Therefor, the one-fold and the two-fold symmetry groups are the only allowable symmetries for the structure.

TE and TM modes profile

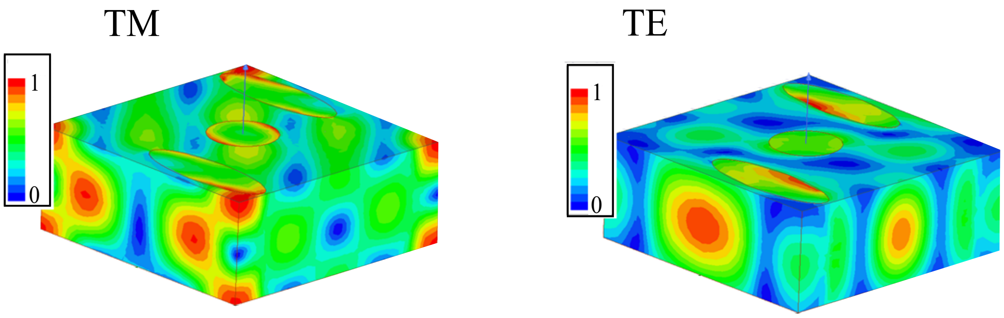

Figure S2: Field profiles associated the degenerate TE and TM modes. The modes cooperatively induce chirality via guided mode resonance. The normalized fields' magnitude are shown.

Optical Characterization Setup

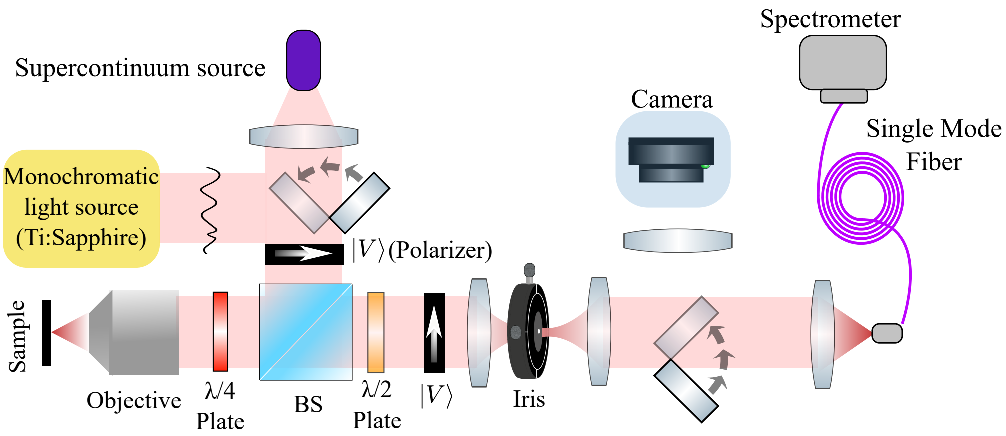

Figure S3: **Confocal microscopy setup:** Reflection spectroscopy is performed by a supercontinuum light source. The beam is passed through a polarizer to purify the polarization (vertical). The beam splitter BS is a non-polarizing splitter which allows us to monitor the two orthogonal states of polarization at the output. The focusing objective has a numerical aperture of $NA = 0.1$ and a working distance of $\sim$ 1cm. We measured the focusing spot size using two-point sharp blade technique. The spot size at the focus is $2w_0 \sim 36\mu$m . The quarter-waveplate right before the objective can be adjusted for right handed or left handed circular polarization incidence when its fast axis is titled by $\theta = \pm 45°$ with respect to the vertically polarized beam. The half-waveplate and the polarizer at the output allow us to monitor the diagonal and off- diagonal elements of the reflection tensor in the circular basis. If fast axis of the half-waveplate is vertical, then the diagonal elements of the reflection matrix are coupled to the output i.e. r_{++} and r_{--}. If the half-wave plate is rotated by the angle of $\theta = 45°$ the off-diagonal elements (r_{+-} and r_{-+}) are coupled to the output. To get rid of background reflections mainly due to the silicon substrate underneath the membrane, we use confocal arrangement (two lenses and the iris in between). After passing through the confocal setup, the beam is coupled to the single mode fiber at the output which further purifies the signal for very clean spectroscopy.

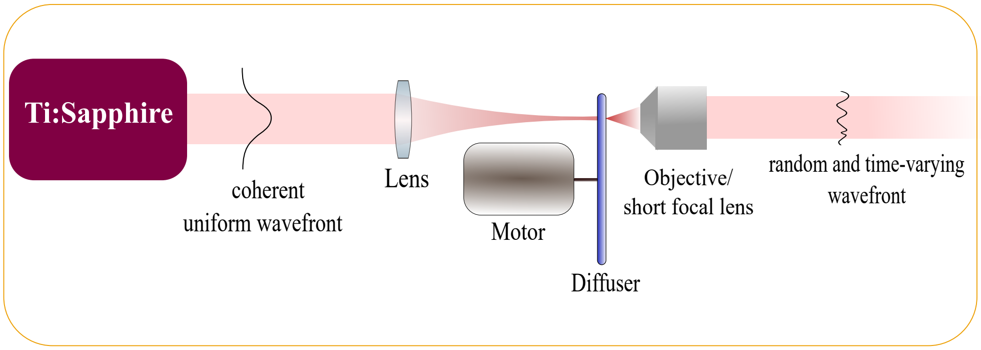

Figure S4: **Monochromatic light source:** A ground glass diffuser is used to destroy the spatial coherence of the laser beam. However, that would mess up with the image quality as the beam becomes completely non-uniform. In order to keep the quality of imaging as high as possible, we rotate the diffuser by means of a stepper motor. If the speed of rotation is much larger than the integration time of the camera, the camera captures a time-averaged distribution of the beam which should be fairly well uniform as the diffuser has random distribution of grit on it.

Measurement Results

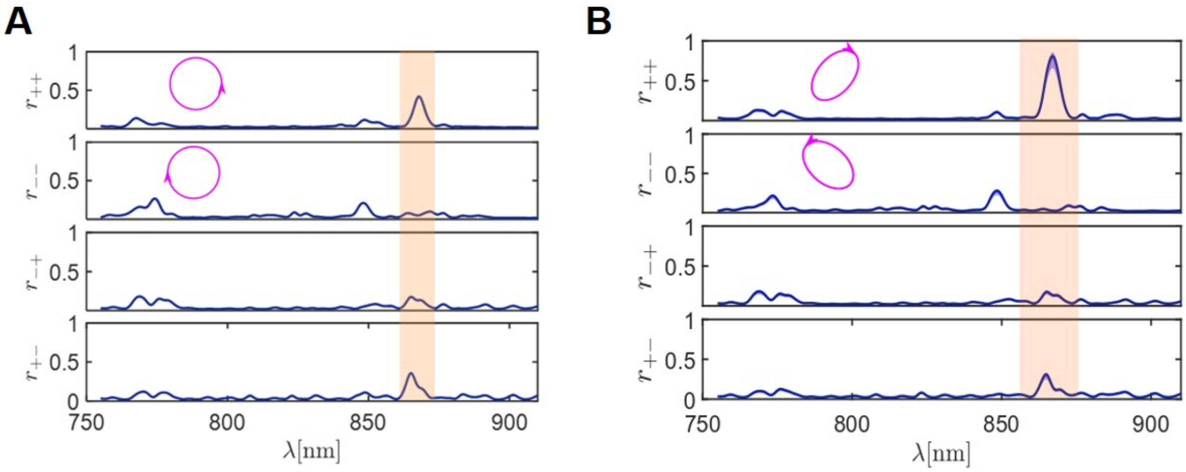

Figure S5: **Elements of the reflection tensor**: (A) purely circularly polarized (B) elliptically polarized eigen-modes.

Sample fabrication: undercut region and zoomed SEM

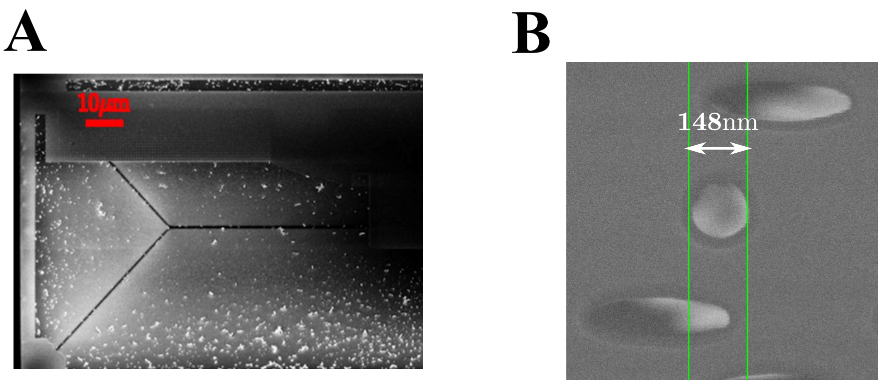

Figure S6: **SEM images** (A) V-shape undercut region. The rays reflecting off the V-shape undercut region are mostly off-normal and therefore they are mostly avoided in confocal spectroscopy (B) Zoomed SEM image of the holes. Due to an imperfect etching the diameters of the holes on the top and the bottom surfaces are slightly different.

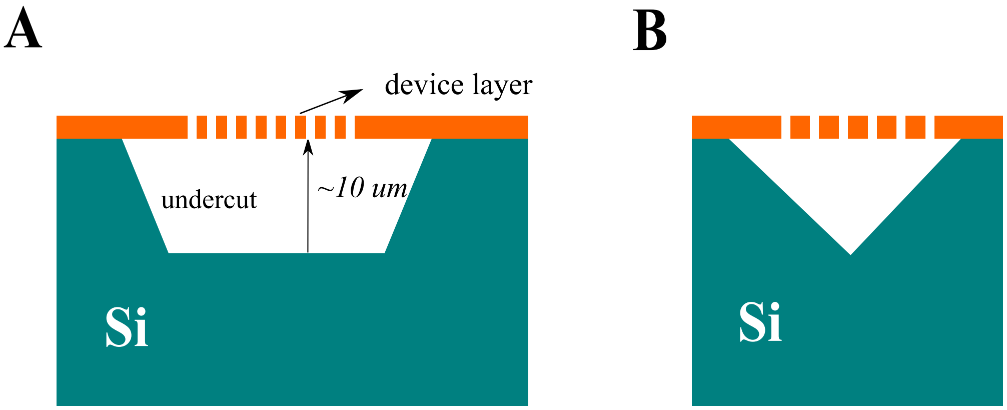

Figure S7: **Schematics of the undercut region** at two side views.

Reflection of Gaussian Beam from the Photonic Crystal Slab

Full-wave simulation of the structure illuminated by a focused Gaussian beam –with a big beam waist– is numerically expensive and does not yield correct results due to accumulation of numerical errors. Instead, hybrid numerical techniques can be effectively employed. To estimate the reflectivity of a Gaussian beam with a beam waist similar to the experimental condition i.e. $w_0 \approx 18\mu m$, we have employed the plane wave expansion technique. Response of the photonic crystal to different plane waves with nonzero tangential wavenumber-denoted by $k_{||}$- has been calculated. The Fourier distribution of the focused Gaussian beam is given by $E(\mathbf{k}_{||}) \approx \sqrt{\pi} w_0 E_0 \exp(-w_0^2 k_{||}^2/4)$ where E_0 is the amplitude of the field at the center of the beam. The reflection coefficients of the obliquely incident plane waves for opposite helicities are shown in Fig. S8. The reflectivity of the Gaussian can be estimated as follows

$$r_{ij} \approx \frac{\int_{-\infty}^{+\infty} dk_{||} \left[r_{ij}(k_{||}) \left| E(\mathbf{k}_{||}) \right|^2 \right]}{\int_{-\infty}^{+\infty} dk_{||} \left| E(\mathbf{k}_{||}) \right|^2} \tag{6}$$

where $r_{ij}(k_{||})$ is the power reflectivity of the obliquely incident circularly polarised plane-wave with the tangential wave number $k_{||}$ (shown in Fig. S8). The subscripts i, j denote the state of polarisation (i.e RHCP or LHCP). According to Eq. (S8), at the deign wavelength of $\lambda_0 = 870$nm, the estimated reflectivity of the RHCP Gaussian beam reaches up to $\sim 80\%$ while for the opposite helicity the reflectivity remains below $r_{--} \lesssim 5\%$.

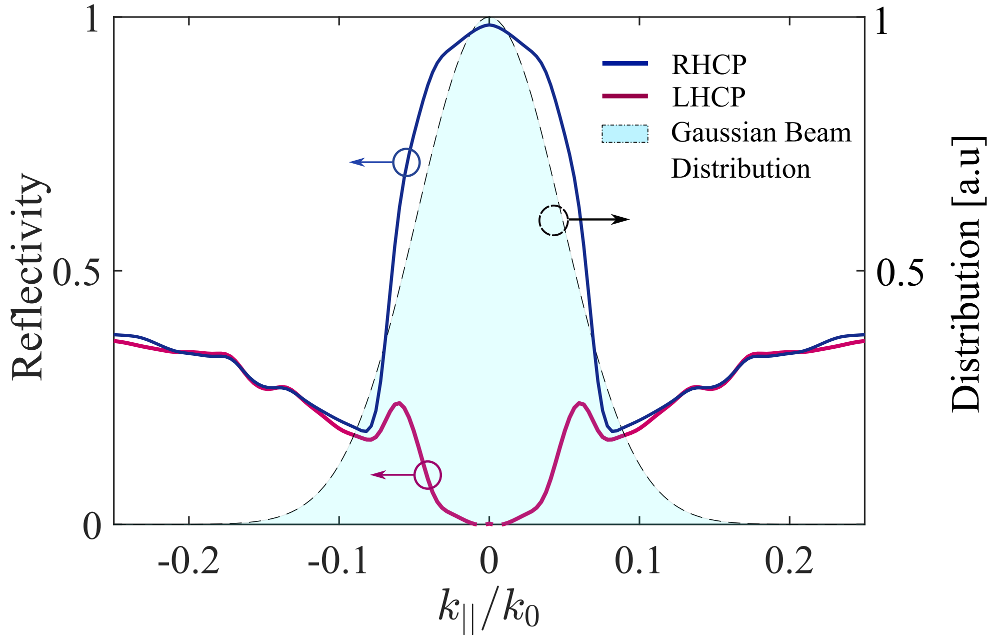

Figure S8: Reflectivity of obliquely incident RHCP and LHCP lights at the design wavelength of $\lambda = 870$nm versus the tangential wave-number $k_{||}$. The blue shaded curve is the normalized Fourier distribution of a focused Gaussian beam with a beam waist of $w_0 = 18\mu m$.

Finite-Size Photonic Crystal Slab

We have performed multiple simulations to observe how many unit cells are required to achieve a noticeable chiroptical effect. Since the proposed photonic crystal is expected to anomalously reflect light into its co-circular polarization, that would be sufficient to monitor the portion of the power reflections from the finite slabs that maintain the handedness. This eliminates the impact of diffraction from a finite size slab and thus enabling us to solely characterize the chiral effect.

It is worth emphasizing that, owing to the symmetry of the unit cells, chirality is only manifested in the spin-preserving terms. We define the *fractional chiral reflectivity* from a finite slab as $\alpha = r_{++}/(r_{++} + r_{-+})$. The fractional reflectivity versus the number of unit cells for square-shape photonic crystals with various sizes is plotted in Fig. S9.

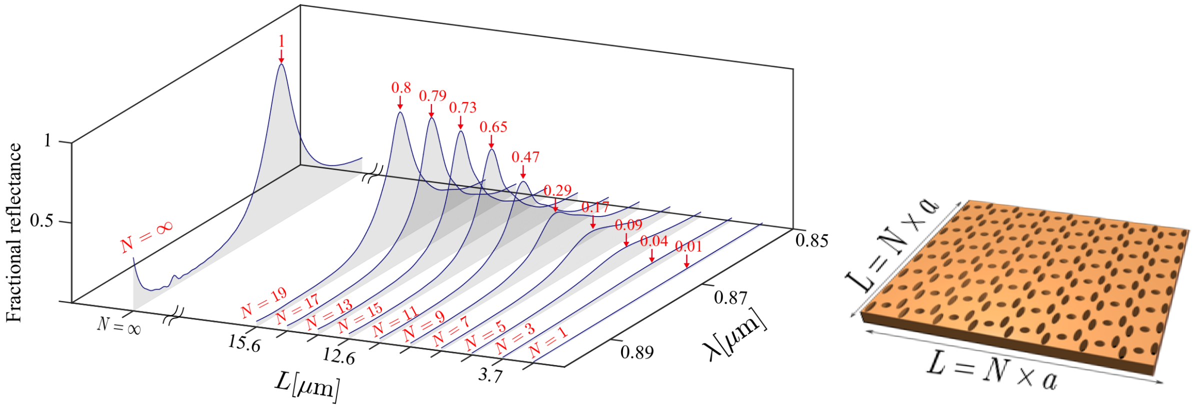

Figure S9: Chirality of a finite size photonic crystal slab. The fractional reflectivity of $\alpha = r_{++}/(r_{++} + r_{-+})$ is plotted for a number of finite-size slabs. N denotes the number of the unit cells at each dimension; the slabs contain $N \times N$ unit cells.

Angle of View

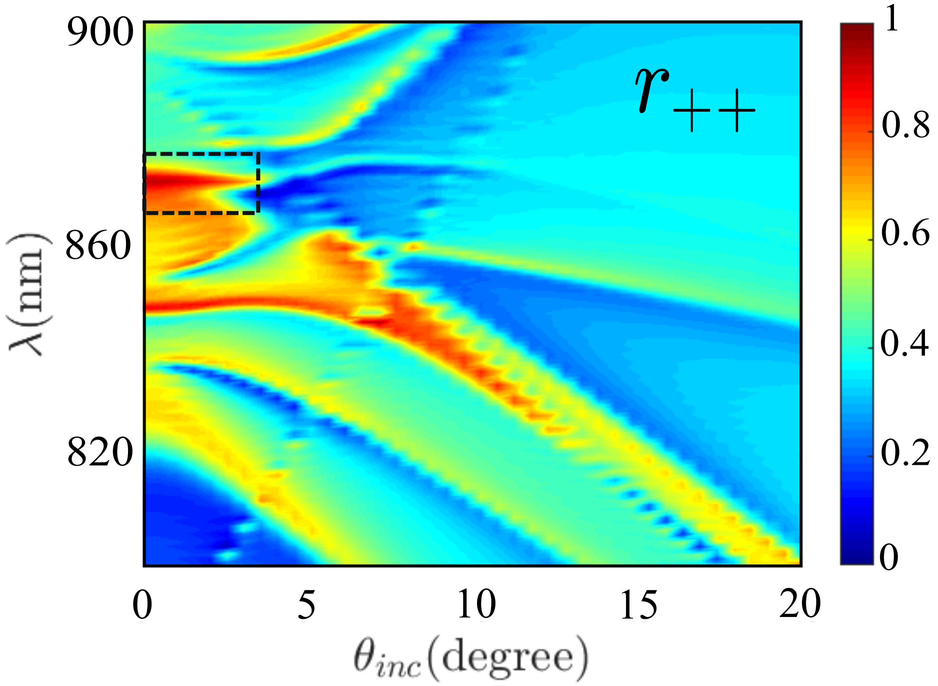

Figure S10: **Angle dependence of the reflection coefficient:** total reflectivity of the chosen helicity. The region signed by the dotted lines, shows the operation band of the chiral mirror. for $\theta \lesssim 5^\circ$ the mirror is reasonably well reflective.
